# Soft Skills and STEM Education: Vision of the European University EURECA-PRO

**DOI:** 10.1007/s00501-022-01275-7

**Published:** 2022-09-13

**Authors:** Ainhoa Villán-Vallejo, Abir Zitouni, Paula García-Llamas, María Fernández-Raga, Adriana Suárez-Corona, Roberto Baelo

**Affiliations:** 1 Department of General and Specific Didactics and Educational Theory, University of León, 24071 Leon, Spain; 2 Department of Applied Physics, University of León, 24071 León, Spain; 3 Department of Mathematical Sciences, Universidad de León, 24071 León, Spain

**Keywords:** EURECA-PRO, STEM, Soft skills, Learning methods, EURECA-PRO, MINT, Soft Skills, Lernmethoden

## Abstract

Science, Technology, Engineering, and Mathematics (STEM) disciplines play an increasingly important role in the current socio-economic context. Higher education systems are working to equip students with the appropriate skills and competencies to cope with current demands and, in particular, to join a labour market strongly informed by STEM disciplines. Many such skills are non-disciplinary and are known as transversal or soft skills. Soft skills, including interpersonal and socio-emotional skills, are highly sought after in the labour market. These skills not only reflect personal abilities but also draw on concepts, such as social responsibility, creativity, ethics, and emotional intelligence. The European University on Responsible Consumption and Production (EURECA-PRO) seeks to foster soft skills development in our activities and curricula.

## Abstract (de)

Mathematik, Informatik, Naturwissenschaft und Technik. (MINT) spielen im aktuellen sozioökonomischen Kontext eine immer bedeutendere Rolle. In diesem Sinne arbeitet das Hochschulsystem daran, die Studierenden mit den geeigneten Fähigkeiten und Kompetenzen auszustatten, um den aktuellen Anforderungen und insbesondere denen eines stark von MINT-Disziplinen geprägten Arbeitsmarktes gerecht zu werden. Viele dieser Fähigkeiten sind nicht fachbezogen und werden als transversale Fähigkeiten oder Soft Skills bezeichnet.

Soft Skills sind auf dem Arbeitsmarkt sehr gefragt, einschließlich interpersoneller und sozio-emotionaler Fähigkeiten. Diese Fähigkeiten sind nicht nur mit rationalen oder persönlichen Fähigkeiten verbunden, sondern umfassen auch andere Aspekte, wie soziale Verantwortung, Kreativität, Ethik und emotionale Intelligenz.

Die Europäische Universität für verantwortungsvollen Konsum und verantwortungsvolle Produktion (EURECA-PRO) ist sich der Notwendigkeit bewusst, Soft Skills in unseren Aktivitäten und Lehrplänen zu verbessern.

## Introduction

Highly-developed countries are building and improving their socio-economic models to reflect important 21st-century skills (e.g., digital competencies, interculturality, critical thinking, and communication)[1]. Concurrent developments in Science, Technology, Engineering, and Mathematics (STEM) disciplines enable this socio-economic model. Simultaneously, such skills require specific training, especially for younger generations. Integrating soft skills with STEM, the Washington STEM Study Group [2, 3] proposed that students are considered STEM-literate when they understand how the world works according to the four STEM disciplines and can apply this understanding to improve social, economic, and environmental conditions in all social spheres.

The European Commission (EC) forecast seven million STEM-sector job openings by 2025. This means that STEM-skilled employees have excellent prospects of employability and professional career development. Given that education attempts to offer appropriate curricula and training to help students in their future professions, promoting STEM is considered a priority in the European educational system. Accordingly, many organisations significantly involve STEM in their activities. This includes promoting STEM in education and training, as well as helping future graduates to develop a range of critical skills/competencies. Examples of such projects and activities are maker labs or spaces, robotics activities, analytical technologies, virtual reality, artificial intelligence, and the Internet of Things (IoT).

However, only 34% of STEM graduates are women [4], and most of these are in the field of science. Due to this lack of female presence in STEM areas, a number of institutions are conducting campaigns and initiatives to increase women’s interest in STEM fields. The European Union (EU) is trying to close this gender gap through several pathways. One such pathway is the development of a gender equality, regulatory framework focused on the labour market and research with three key goals: gender parity in research and innovation, gender equality in careers, and gender balance in decision-making bodies. Such initiatives highlight the importance of women’s presence in STEM fields. In addition, EU-supported programmes include the European Platform of Women Scientists (EPWS), the European Association for Women in Science, Technology, Engineering, and Mathematics (WITEC), the European Scenery on Gender and STEM (SESTEM: Supporting Equality in Science Technology and Mathematics), the Gender Equality Network in the European Research Area (GENERA), and the project GenSET, focused on gender action plans in science, engineering, and technology. As a result of these and many other initiatives, the presence of women in STEM fields has increased in the last few years. These efforts must continue to grow.

Over the decades, a profound process of specialization in the scientific and academic fields as well as in the labour market has occurred. However, in recent years, the labour market has increasingly come to recognise and demand soft skills as a criterion for employability. Academia must therefore likewise shift in this direction. Skills such as leadership, creativity, communication, management, professionalism, ethics, agility, resilience, and flexibility are some examples of soft skills that are standard professional requirements in the 21st century.

Professional and governmental organisations have taken steps to promote the acquisition of soft skills. However, from a broader perspective, there is still a lack of understanding of the substantial impact these skills have in STEM-field careers [1]. Campos et al. [1], in analysing how the labour market requires soft skills, drew attention to the results of OECD [5–7] studies showing that desired employability skills include reasonable text interpretation, mathematical knowledge, information management, and problem-solving skills. Students and companies [8] consider that, in the last years, there has been an increase in consideration of soft skills as desirable employability skills face technical skills. In addition, this study [8] highlighted the relevance of students’ proactive role in developing soft skills to enhance their employability.

Higher education institutions offer opportunities to acquire soft skills through various initiatives. In addition, they offer the opportunity to participate in different activities and projects hosted both by higher education institutions and by external organisations that support women in science, technology, art, engineering, and mathematics. Various research studies have been conducted to examine and understand why women should join STEM areas and how soft skills can help women stand out in these fields. Such an understanding will help to reduce the gender gap common in STEM. One study shows that the implementation of female mentors to increase female role models in STEM fields helps women to have a positive outlook on theses professions [9]. A study conducted with 20 female engineering and technology students in Poland by Zawistowska [10] showed that having a significant female model in their close social network active in the STEM fields influenced participants’ own decision to choose such a stereotypically masculine field. Another example is the “Entra 21 program” implemented in 18 Latin American countries that includes a soft skills training component. Although there is no rigorous evaluation of this intervention, employers report that program participants have a remarkable ability to work better in teams and to take on increased responsibility as compared to other employees [11]. Finally, female authors like Janice Koch, Barbara Polnick, and Beverly Irby, editors of GIRLS and Women in STEM: A Never-Ending Story [12], share stories of successful women in STEM to showcase how women’s integration in these fields increases innovation, creativity, problem-solving, and teamwork. A book that is accessible to readers and “can serve as a basis for beginning the disclosing students, teachers, and scholars” [13].

Such programs and trainings help immensely to harness women’s soft skills in STEM fields and may lead to more effective problem solving and improved innovation [14]. Reinforcing soft skills gives women a better chance of becoming integrated into STEM fields.

## Purpose

The European University on Responsible Consumption and Production (EURECA-PRO) seeks to promote soft skills to improve our students’ employability. By doing so, we seek to support STEM students, upgrade their training and employability skills, encourage lifelong learning and participation in mobility programs, and promote internationalisation and interculturality. To this end, we have designed courses, conferences, colloquiums, and many other activities that nurture the development of soft skills. In addition, academic programs in which active learning methodologies, cross-cultural, and soft skills are fully integrated into curricula are currently being developed.

Promoting STEM education presents challenges for EURECA-PRO as well. A few of these challenges are questions of how to embrace a holistic perspective on STEM competencies, how to use soft skills as a tool to increase female interest in STEM fields, and ultimately how to increase the number of women with STEM vocations.

### EURECA-PRO: STEM and Women, Soft Skills and Active Learning Methodologies

STEM improves human mind, intellectualism, critical thinking and rational thoughtfulness development [15].

A STEM education approach aims at integrating the fields of science, technology, engineering, and mathematics and their associated practices and skills starting at an early age. In doing so, STEM education focuses on the teaching process so that the student will develop a set of cross-curricular capacities to be used as valuable tools with which they can build the foundation for a chosen, future career. Unfortunately, women have traditionally been underrepresented in STEM fields [16]. This underrepresentation can be traced back to two influential factors: first, young women lack role models that demonstrate their STEM-related possibilities, and second, a lack of specific STEM training that fails to awaken talent in these areas that might otherwise be overlooked due to traditional socialisation.

As previously stated, several entities have launched different activities to increase women’s presence in the STEM fields. Many of these efforts can be tied to an increasing number of women in STEM studies or specific STEM activities in recent years [17]. Developing a career in science, technology, engineering, mathematics, or in any other field takes years, during which the necessary abilities and knowledge are acquired. STEM companies are increasingly looking for ever-more prepared profiles replete with technical skills and expertise, including interpersonal and intrapersonal skills known as core or soft skills.

Several studies conclude that women show high leadership capacities, creativity, and resilience in their workplaces. In addition, recent research has revealed the necessity of emphasizing the use of creativity and design in attracting girls to STEM academic and career fields [18–20]. Such skills not only help women in STEM careers, they inspire other girls to pursue such paths. In this sense, soft skills could contribute to developing women’s interests in STEM fields and thereby play an important role in closing the gender gap that has existed for so long.

Soft skills complement disciplinary knowledge and are required in all professions. These skills include personal abilities that improve performance (such as problem-solving or critical thinking), the ability to facilitate personal and professional interactions (by being able to speak publicly, identify written and unwritten business rules and work culture), teamwork and leadership skills, work ethic, intercultural fluency, and digital literacy. According to the McKinsey Global Institute [21], the rapid introduction of technological development emphasises the need for workers to develop suitable soft skills; companies increasingly identify a lack of appropriate skills as a barrier to realising the possibilities and benefits of automation and technology. Therefore, self-management skills and the ability to reskill are highly valuable to employers. In fact, according to the World Economic Forum, which has been tracking employers’ skill demands since 2016, cross-functional (soft) skills such as critical thinking and problem solving have been considered top skills year after year. As reflected in their “Future of Jobs report 2020” [22], the need for these abilities is only expected to increase.

Furthermore, the global working reality demands these soft skills. The results of Erasmus+ Higher education Impact study [23] can help in understanding this demand. In it, a survey of 77,000 participants showed that 72% of participants considered the mobility program experience as beneficial for increasing their technical, interpersonal, and intercultural skills and competencies as well as their self-confidence, their ability to achieve goals, and their social and cultural openness. Furthermore, 80% of Erasmus graduates found a job within three months of their graduation due to their mobility experience.

EURECA-PRO is committed to promoting employability. To do so, EURECA-PRO’s TransPlat platform offers several free, online training courses that improve soft skills such as communication, team-building, digital skills, cybersecurity, languages, and intercultural competencies. In addition, active learning methodologies such as Project Based Learning (PBL), cooperative learning, and courses promoting a healthy lifestyle and habits help to support the future employability of EURECA-PRO students.

In the field of STEM education, the EURECA-PRO alliance has also organised initiatives such as the “STEM Innovation Contest” that gathered several interdisciplinary, EURECA-PRO teams to address real-world problems proposed by European, STEM-related companies. This challenge aimed to promote innovation and problem-solving skills through student collaboration on creative projects. In addition, the contest connected students, teachers, and staff from various EURECA-PRO universities and thereby created job opportunities, made changes to European education by highlighting and solving educational program challenges, promoted mobility, diversity, and gender equality, and encouraged women to join STEM fields. Additionally, EURECA-PRO designed the “Women in STEM Colloquium” to take place on the International Day of Women and Girls in Science in European secondary schools to encourage girls to join STEM fields. In this colloquium, female, lead EURECA-PRO researchers in STEM fields shared their STEM career paths and discussed what different fields have to offer. By sharing themselves as potential role models for a successful STEM career, these researchers showed young students the possibilities of a professional STEM path.

Training courses and the variety of programs offered by the EURECA-PRO alliance are key to providing students with the appropriate tools to access jobs with good working conditions where their contributions will be valued. All these actions have led to positive results and achievements. Within the context of the COVID-19 pandemic, the demand for courses that help employees with personal development, well-being, and health when managing remote and hybrid work has increased significantly. This increases the need to upskill and reskill employees in self-managing skills such as stress tolerance, resilience, and flexibility. EURECA-PRO will continue to work on transforming education and preparing students for the labour market while promoting sustainability through responsible consumption and production.

## Reflections

The promotion of STEM disciplines and their related skills has been a goal of EURECA-PRO and the European Commission (EC), as well as of international organisations such as the United Nations (UN). This is evidenced by the UN’s inclusion of job skills and future sustainable employability as part of the Sustainable Development Goals (SDGs) within the framework of their 2030 Agenda. A STEM education approach aims at integrating the fields of science, technology, engineering, and mathematics and their associated practices and skills starting at an early age. In doing so, STEM education aims to focus on the teaching process and on the students to help them develop a set of cross-curricular capacities that will provide the valuable tools necessary to build the foundation for any chosen, future career. Whether it is a career in science, technology, engineering, mathematics, or in any other field, acquiring the necessary abilities and knowledge takes years.

The promotion of women in STEM fields reflects the importance of balanced gender presence in these disciplines. It encourages young girls to join science, technology, engineering, and mathematics careers. Women have joined forces by establishing various associations and organizations (e.g., Mothers of Innovation, Wise Women in Technology, and Girls in Tech) that support gender equality in STEM. Higher education, in particular, is considered the primary method to reduce the gender gap in the STEM fields by offering diverse, challenging, and exciting topics that valorise the status of women in these disciplines.

EURECA-PRO has developed successful programs in the development of soft skills; this has required the active and continuous cooperation of all member universities, researchers, and staff to create projects, courses, activities, and trainings that promote the importance of soft skills, the use of active learning methods, the value of STEM education, and the importance of supporting women in STEM. The success of these programs and activities lies in their multidisciplinarity, internationalisation, and practicality. In addition, such programs unfailingly foster sustainability through responsible consumption and production.
